# Advancing the implementation of artificial intelligence in regulatory frameworks for chemical safety assessment by defining robust readiness criteria

**DOI:** 10.3389/frai.2025.1738770

**Published:** 2026-01-20

**Authors:** Joyce de Paula Souza, Jonathan Blum, Uko Maran, Sulev Sild, Louis Dawson, Aleksandra Čavoški, Laura Holden, Robert Lee, Veronika Karnel, Lukas Meusburger, Sandrine Fraize-Frontier, Alexander Walsh, Gilles Rivière, Giuseppa Raitano, Alessandra Roncaglioni, Emma Di Consiglio, Olga Tcheremenskaia, Cecilia Bossa, Lina Wendt-Rasch, Tomasz Puzyn, Ellen Fritsche

**Affiliations:** 1 Swiss Centre for Applied Human Toxicology, Basel, Switzerland; 2 Department of Pharmaceutical Sciences, University of Basel, Basel, Switzerland; 3 Institute of Chemistry, University of Tartu, Tartu, Estonia; 4 Birmingham Law School, University of Birmingham, Birmingham, United Kingdom; 5 Department Risk Assessment, Austrian Agency for Health and Food Safety (AGES), Vienna, Austria; 6 French Agency for Food, Environmental and Occupational Health and Safety (ANSES), Maisons-Alfort, France; 7 Department of Environmental Health Sciences, Mario Negri Institute for Pharmacological Research, Milan, Italy; 8 Environment & Health Department, Italian National Institute of Health (ISS), Rome, Italy; 9 Swedish Chemicals Agency (KEMI), Stockholm, Sweden; 10 Faculty of Chemistry, University of Gdańsk, Gdańsk, Poland; 11 QSAR Lab Ltd., Gdańsk, Poland

**Keywords:** artificial intelligence, chemical risk assessment, chemical safety assessment, readiness criteria, regulatory frameworks, regulatory science

## Abstract

The integration of artificial intelligence (AI) into chemical risk assessment (CRA) is emerging as a powerful approach to enhance the interpretation of complex toxicological data and accelerate safety evaluations. However, the regulatory uptake of AI remains limited due to concerns about transparency, explainability, and trustworthiness. The European Partnership for the Assessment of Risks from Chemicals (PARC) project *ReadyAI* was established to address these challenges by developing a readiness scoring system to evaluate the maturity and regulatory applicability of AI-based models in CRA. The project unites a multidisciplinary consortium of academic, regulatory, and legal experts to define transparent and reproducible criteria encompassing data curation, model development, validation, explainability, and uncertainty quantification. Current efforts focus on identifying key priorities, including harmonized terminology, rigorous data quality standards, case studies, and targeted training of regulatory scientists. *ReadyAI* aims to deliver a practical, evidence-based scoring system that enables regulators to assess whether AI tools are sufficiently reliable for decision-making and guides developers toward compliance with regulatory expectations. By bridging the gap between AI innovation and regulatory applicability, *ReadyAI* contributes to the responsible integration of AI into chemical safety assessment frameworks, ultimately supporting human and environmental health protection.

## 1 Introduction

Recent advances in artificial intelligence (AI), encompassing machine learning and deep learning, are leading to a new era of data-driven innovation. These computational approaches are increasingly recognized as powerful tools across numerous disciplines. In regulatory toxicology, a field dedicated to protecting human health from adverse effects of chemical exposure, AI might offer significant support. This is especially true given the need to evaluate large volumes of datasets in the chemical risk assessment (CRA) process. Several recent publications highlighted the potential application of AI in safety assessment (Wittwehr et al., 2020; Lin and Chou, 2022; Wassenaar et al., 2024; Hartung and Kleinstreuer, 2025), emphasizing the importance of building trust in these technologies to facilitate their regulatory implementation into CRA. As a cornerstone of public health protection, CRA assesses chemical risk by integrating toxicological hazard data and exposure scenarios, within a framework of regulatory requirements. Its goal is to prevent environmental and human health adverse outcomes (e.g., acute/chronic diseases or developmental and reproductive disorders) associated with chemicals present in the environment or food (e.g., contaminants) or used in consumer products, pharmaceuticals, agriculture, and industrial processes.

The CRA process is currently undergoing an international paradigm shift moving from relying on endpoint measurements in animal studies toward human-relevant, mechanism-based, animal-free, and data-driven, integrating testing approaches using New Approach Methodologies (NAMs) (Schmeisser et al., 2023). This evolution entails not only the adoption of novel data generation techniques but also the development of fit-for-purpose frameworks for data evaluation, integration, and analysis. Within these frameworks, all available data are considered, provided they meet defined quality and validity criteria. However, the manual identification, retrieval, evaluation, and curation of such data are highly time-consuming. As a result, AI-driven computational approaches in CRA are increasingly recognized as powerful tools for various applications in toxicology, including quantitative structure–activity relationships (QSARs), exposure modeling, read-across, systematic reviews, and evidence integration for adverse outcome pathways development. Supported by AI, researchers, safety assessment scientists, and regulatory authorities, could work toward increasing accuracy, efficiency, time- and cost-effectiveness of assessment practices while decreasing reliance on *in vivo* animal testing (Blümmel et al., 2024). The latter aligns with worldwide trends on animal experimentation phaseout, announced by the European Commision (2025), United States Food and Drug Administration (2025), and United States National Institutes of Health (2025).

Similarly to several NAMs, the regulatory uptake of AI in toxicological sciences remains limited; there is no established regulatory requirements for incorporating AI-based tools into CRA, despite its growing number of potential use cases. The responsible regulatory uptake of AI is a topic of international discussion, supported by initiatives such as the Global Summit on Regulatory Science and the Global Coalition for Regulatory Science Research (2025). The enthusiastic discussion around the implementation of AI in CRA is now sharing space with debates regarding the uncertainties and transparency of AI. The integration of AI into regulatory frameworks, supporting regulatory decision-making, requires that these systems demonstrate trustworthiness, explainability and reliability. Moreover, regulatory scientists need adequate guidance and training to understand the principles, benefits, and limitations of AI algorithms. This ensures that regulatory scientists can critically evaluate AI-generated outputs and make well-informed decisions based on them. Ultimately, achieving a balance of human-AI interaction, responsibility, and accountability will be crucial for the successful integration of AI into regulatory decision-making (Hartung et al., 2025).

**Figure 1:**
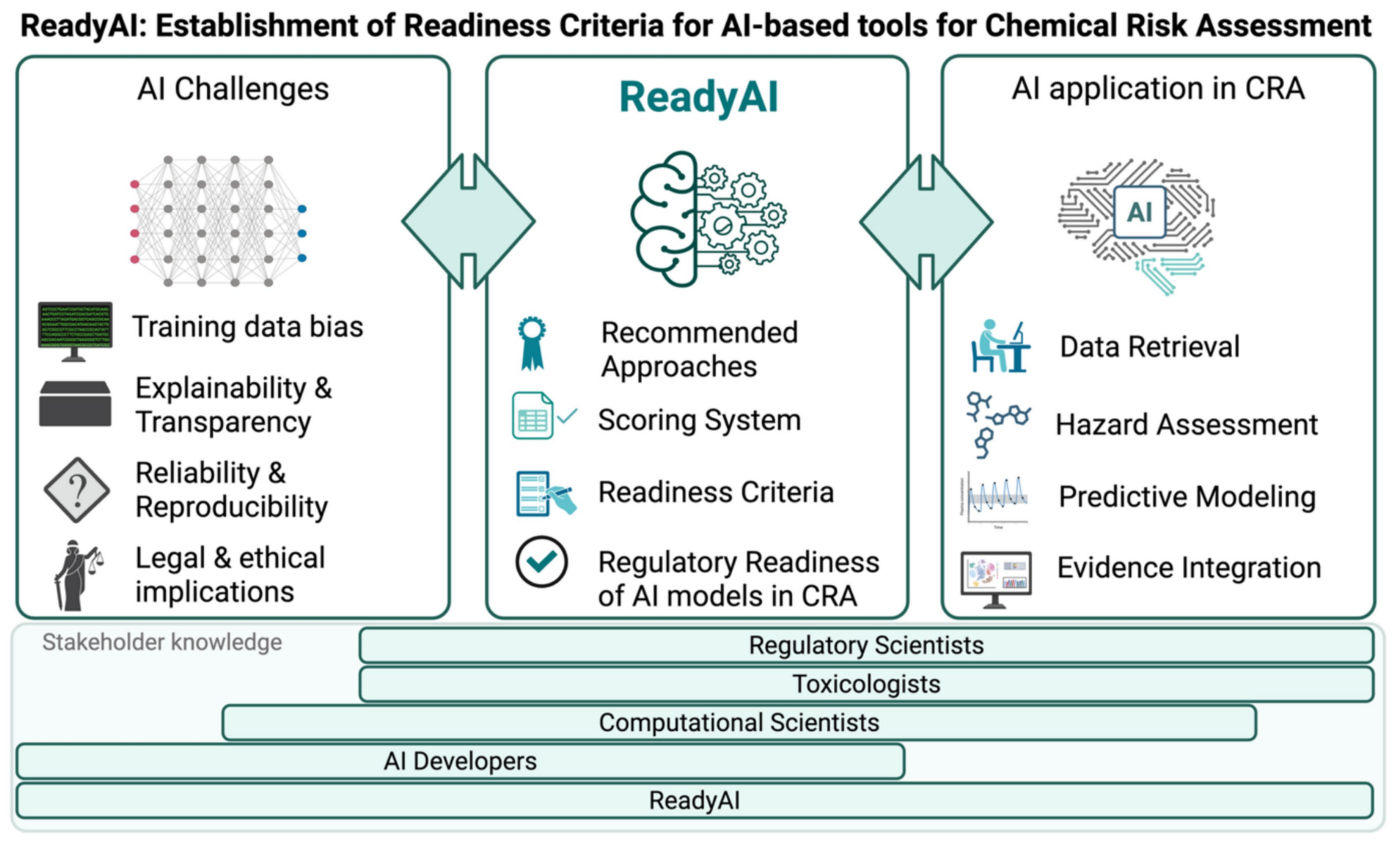
*ReadyAI* aims to bridge the gap between AI development and regulatory application by defining an AI scoring system that enables regulatory scientists to assess the readiness, reliability, and applicability of AI-based models for chemical risk assessment. The project will promote structured communication among key stakeholders, including AI developers, researchers, computational scientists, regulatory scientists, as well as regulatory authorities. AI, Artificial intelligence; CRA, Chemical risk assessment. Figure was created in BioRender. Swiss Centre for Applied Human Toxicology (2025) https://BioRender.com/96ine4s.

In this context, and as part of the European Union (EU) Partnership for the Assessment of Risks from Chemicals (PARC) initiative (Work Package 6, project P6.4.2.e), we initiated the project *ReadyAI: Establishment of Readiness Criteria for AI-based Tools for Risk Assessment* (Project lead: University of Basel, Switzerland & University of Gdańsk, Poland). This project aims to tackle key challenges in implementing machine- and deep learning in regulatory frameworks by developing a comprehensive scoring system to evaluate the development, training, validation, and maintenance of AI-based tools for use in CRA. To define these criteria, *ReadyAI* aims to foster effective communication between regulatory scientists, regulatory authorities, and AI developers. The resulting scoring system is intended to inform users about the readiness and associated uncertainties of AI-based tools, thereby enabling an objective assessment of their regulatory applicability (Figure 1).

## 2 Launch of the *ReadyAI* project

The *ReadyAI* project was officially launched with the kick-off meeting (May 19th, 2025, in Vienna, Austria), bringing together 21 partners from 9 institutions, including academia and regulatory authorities. This multidisciplinary group convened to align on the overarching goal of the project: *the development of readiness criteria and a scoring system to assess the regulatory applicability of AI tools intended for use in CRA*. The project aims at providing a foundational blueprint to integrate AI into regulatory processes in a scientifically robust, transparent, and responsible manner.

Regulatory readiness refers here to the extent to which an AI tool can be reliably applied within a decision-making process, in accordance with the constraints and requirements of chemicals safety regulation. Drawing inspiration from the established readiness framework for *in vitro* developmental neurotoxicity methods (Bal-Price et al., 2018; Blum et al., 2025) and quantitative structure–activity relationship (QSAR) (Gissi et al., 2024), *ReadyAI* aims to adapt and expand these concepts, creating a tailored scoring system for AI algorithms applied in CRA. The practical applicability of this envisioned scoring system will be assessed through a series of case studies, to be defined in alignment with regulatory stakeholders, which will be conducted over the duration of the project.

The kick-off meeting began with a session on the importance of building trust in AI for regulatory use. Presentations covered foundational concepts and applications of AI in CRA, including data extraction, QSAR quality evaluation, and AI development lifecycle steps (problem identification; data collection and processing; model training, selection, validation, deployment, and maintenance). Critical AI challenges such as data bias, adversarial attacks, and hallucinations were highlighted. The *ReadyAI* project objectives were outlined, with a focus on supporting technical training for regulatory scientists and developing a transparent and reproducible scoring system to assess the readiness and reliability of AI models used in CRA. The session also included a discussion of global regulatory perspectives, noting the current lack of overarching frameworks in some regions including the United States, and the United Kingdom, which adopt a less rigid, pro-innovation approach, as contrasted with the AI Act of the EU (European Commision, 2024b), which classifies AI systems according to risk levels. Within the EU, AI for CRA would likely fall under the high-risk category owing to its potential to impact human health and the environment. Legal scrutiny is therefore vital to the aims of the *ReadyAI* project.

The second part of the meeting focused on European institutional initiatives applying AI in CRA. Ongoing AI initiatives where the Joint Research Centre (JRC) is involved were presented, including the Designathon initiative by the European Partnership for Alternatives to Animal Experimentation (EPAA) (European Commision, 2024a), the Generative AI for Read-Across (GARA) initiative, and AI4AOP (Wittwehr, 2024). Other contributions showcased the integration of AI into systematic reviews, dossier completeness checks, and model-based hazard assessment within the European Food Safety Authority (EFSA) and the European Chemicals Agency (ECHA).

The closing discussion addressed critical factors for successful AI integration, including data curation, data quality assessment, model validation, transparent documentation, and clear communication of model strengths and limitations. The need for targeted training of regulatory scientists was emphasized, along with the importance of using precise and harmonized language when describing AI tool functionality and constraints. Participants also highlighted the risk of overcomplicating models, noting that algorithmic complexity does not always yield better performance. A strong consensus emerged on the necessity of high-quality training data to reduce bias and prevent overfitting.

The meeting concluded with a commitment to establishing a multi-agency, cross-sectoral steering committee and the delivery of a detailed project roadmap in 2025, which will guide the activities of the initiative through to its completion in 2029.

## 3 Perspectives for the establishment of regulatory readiness criteria for AI models

The integration of AI, ranging from classical machine learning models to advanced generative AI technologies, into regulatory frameworks requires a structured and transparent approach to evaluate whether AI tools are sufficiently mature, trustworthy, and fit for purpose. Although AI is rooted in computational sciences, many aspects of algorithm development and application rely on expert judgment rather than defined gold-standard rules. The establishment of regulatory readiness criteria for AI tools applied in safety assessment is, therefore, a critical step in bridging the gap among AI innovation, governance, and regulatory acceptance and implementation.

While the CRA is the primary focus of *ReadyAI*, the AI underlying principles and computational models have broad applicability. Therefore, we envision an intersectoral approach for our scoring system, supporting regulatory AI assurance across other domains such as preclinical safety and healthcare, where transparency, robustness, and accountability are equally critical. In line with that, the steering committee of *ReadyAI* has members of Swissmedic, EFSA, ECHA, JRC, and the Organization for Economic Co-operation and Development (OECD), complemented by an academic AI expert.

Our readiness scoring system will be grounded in core principles adapted from the OECD QSAR Assessment Framework (QAF) (Gissi et al., 2024), and extended to address the broader range of challenges presented by AI-based tools. While the QAF provides a well-established structure for evaluating model validity, transparency, and applicability, *ReadyAI* aims to broaden this approach so that it can be applied not only to QSAR models but also to other types of AI models used in CRA. This broader applicability requires expanding QAF key elements to capture aspects that are not traditionally part of QSAR evaluation. We anticipate that the scoring system will incorporate weighted criteria, acknowledging that certain elements carry greater regulatory relevance than others. Weighting will support proportional scoring and allow the system to distinguish between essential prerequisites and more context-dependent quality indicators. By doing so, the readiness scoring system will offer a unified, transparent framework that could support regulatory assessment of a variety of AI models, while maintaining coherence with established regulatory principles.

We envision that the scoring system will be built on some foundational pillars: data curation, overfitting, explainability, and uncertainty quantification.

AI performance relies on high-quality, well-curated training data, which affects bias, overfitting, and accuracy. Thus, data curation should be assigned substantial weight within the scoring system, as it directly impacts the quality and reliability of AI-based models.

Overfitting occurs when an AI model captures noise in the training data rather than learning generalizable patterns, leading to poor performance on unseen data. To achieve a high readiness score, developers must demonstrate the application of robust strategies to prevent overfitting.

Moreover, regulatory decision-making, particularly in contexts involving public health and safety, requires a clear understanding of how and why a model generates its outputs. As such, explainability is essential for regulatory acceptance and should be a key component of the scoring system.

Finally, regulatory scientists need to assess not only the output generated by the model but also its confidence in those predictions. Thus, precise description and quantification of uncertainty will enhance model transparency, foster trust, and ultimately support a more reliable and robust readiness assessment.

Together, these elements outline the conceptual foundations of the *ReadyAI* scoring system and illustrate how it aims to balance scientific rigor with regulatory applicability. As the project progresses, these components will be refined in consultation with regulatory stakeholders to ensure that the final framework is both scientifically robust and readily applicable in real-world regulatory contexts.

## 4 Conclusion remarks

The ambition of *ReadyAI* is to bridge the gap between AI innovation and regulatory usability and acceptance. By defining readiness levels aligned with scientific maturity and regulatory requirements, we aim to provide regulators with a practical tool to evaluate the applicability, limitations, and trustworthiness of AI models applied in CRA. In addition, the outcomes of this project will help AI developers and users, providing clear criteria that must be met for potential regulatory applicability. This will facilitate collaboration between developers and regulators to ensure responsible deployment of AI-methods.

To transition from animal-based safety assessment to the integration of NAMs, including algorithms and AI in regulatory processes, a governance structure and an intersectoral approach are essential.

The integration of AI into regulatory frameworks demands human oversight, responsibility, accountability, technical expertise, and traceability. While regulatory science is still evolving in this area, the successful uptake of AI will ultimately depend on our ability to translate technical excellence into trusted, interpretable, and actionable approaches that align with the high standards of human and environmental health protection and policymaking.

## Data Availability

The original contributions presented in the study are included in the article/supplementary material, further inquiries can be directed to the corresponding authors.
